# Corrigendum: Neuronal IFN-beta–induced PI3K/Akt-FoxA1 signalling is essential for generation of FoxA1^+^T_reg_ cells

**DOI:** 10.1038/ncomms15792

**Published:** 2017-05-22

**Authors:** Yawei Liu, Andrea Marin, Patrick Ejlerskov, Louise Munk Rasmussen, Marco Prinz, Shohreh Issazadeh-Navikas

Nature Communications 8: Article number: 14709; DOI: 10.1038/ncomms14709 (2017); Published 04 24 2017; Updated 05 22 2017

The affiliation details for Marco Prinz are incorrect in this Article. The correct affiliation details for this author are given below:

Institute of Neuropathology, Faculty of Medicine, University of Freiburg, Freiburg 79106, Germany.

BIOSS Centre for Biological Signalling Studies, University of Freiburg, Freiburg 79106, Germany.

