# Supplementary material for: Neuronal IFN-beta–induced PI3K/Akt-FoxA1 signalling is essential for generation of FoxA1+Treg cells
Source: Nat Commun. 2017 Apr 24;8:14709. doi: 10.1038/ncomms14709 (PMC5413980; doi:10.1038/ncomms14709)
Supplement: Supplementary Information — Supplementary Figures. [file ncomms14709-s1.pdf]

Supplementary figure 1

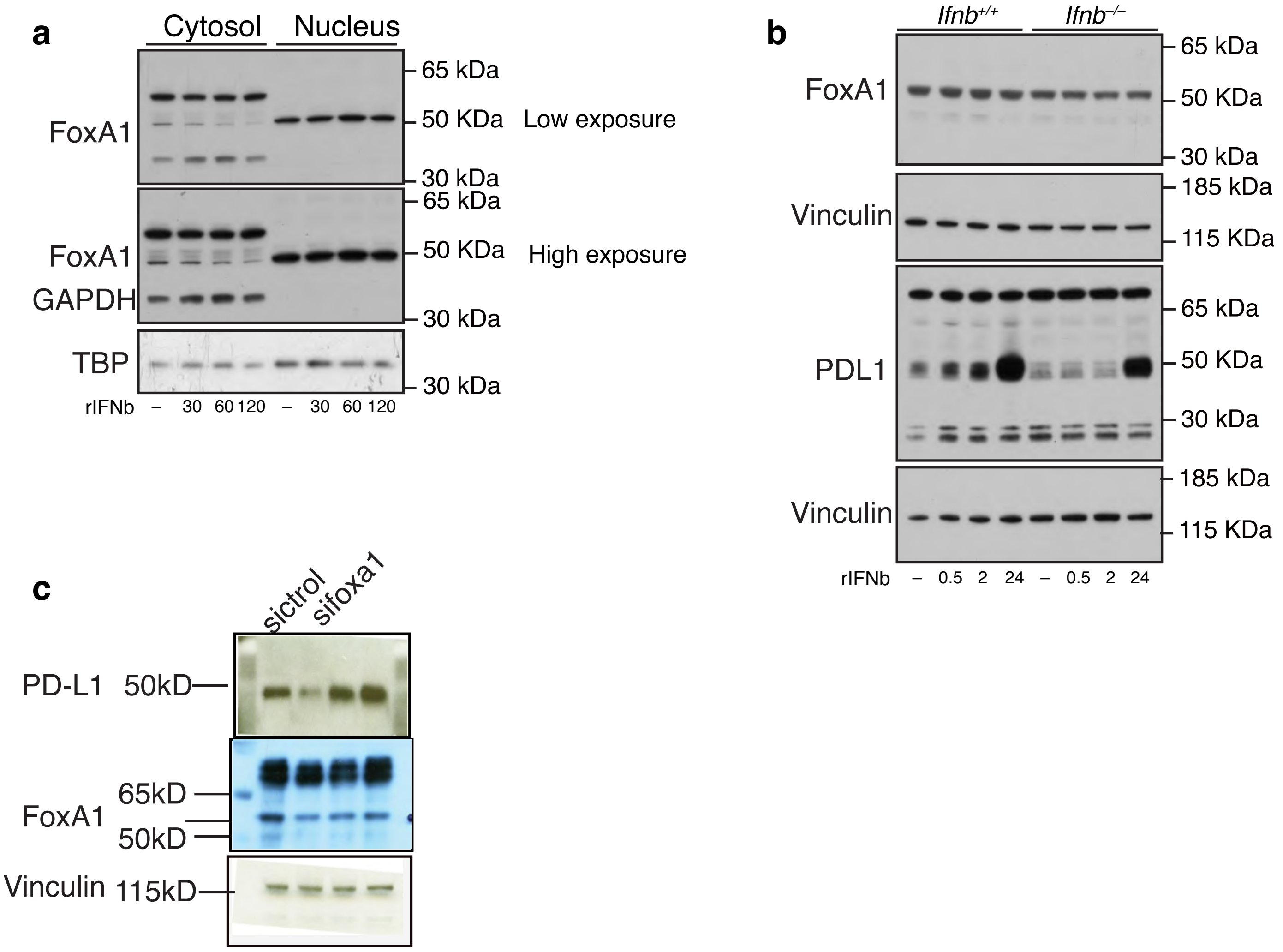

Supplementary figure 1: whole images of western blots in fgure 4

a) Figure 4f **b)** Figure 4h **c)** Figure 4j

Supplementary figure 2

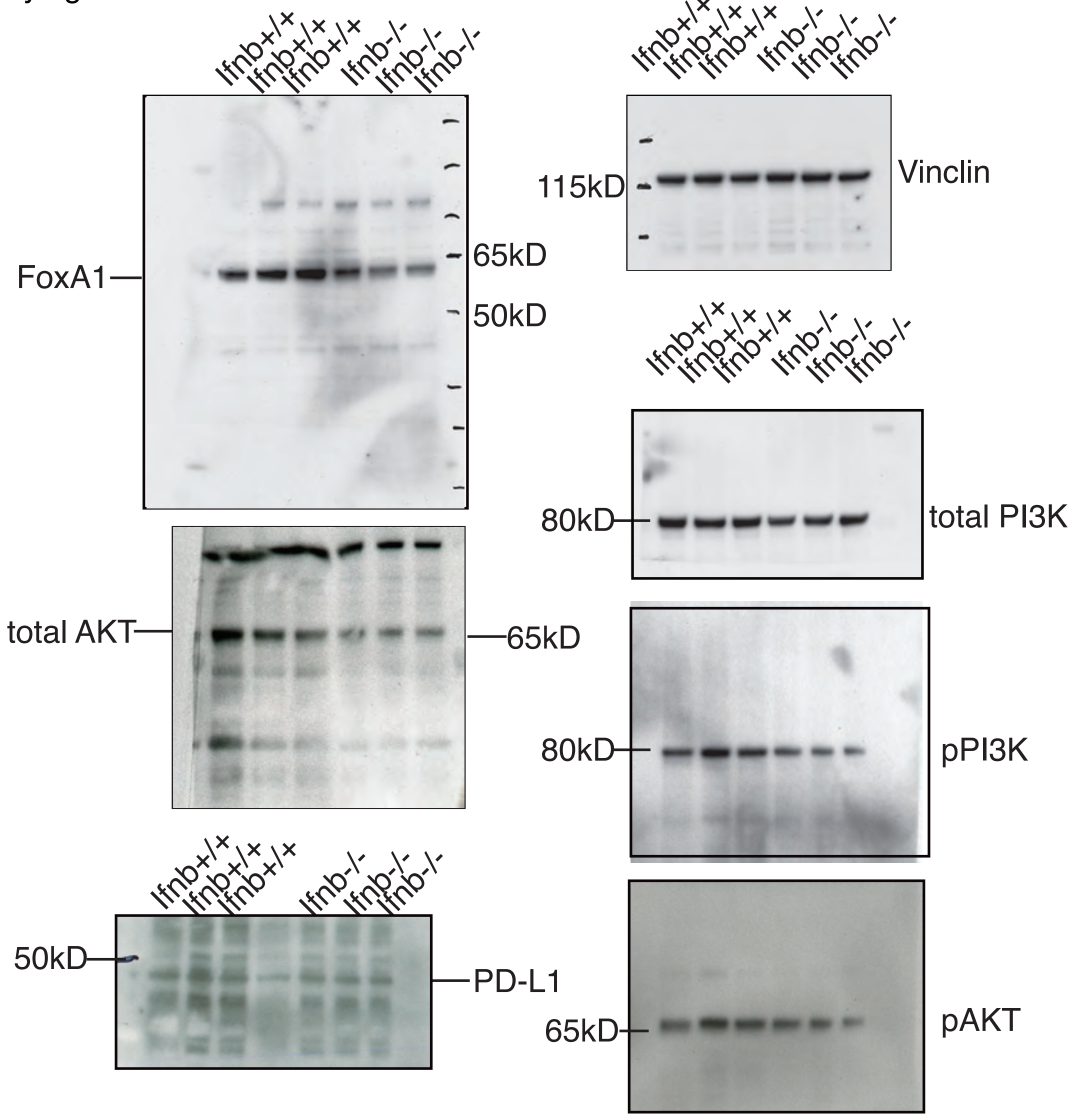

Supplementary figure 2: whole images of western blots in fgure 5b

Supplementary figure 3

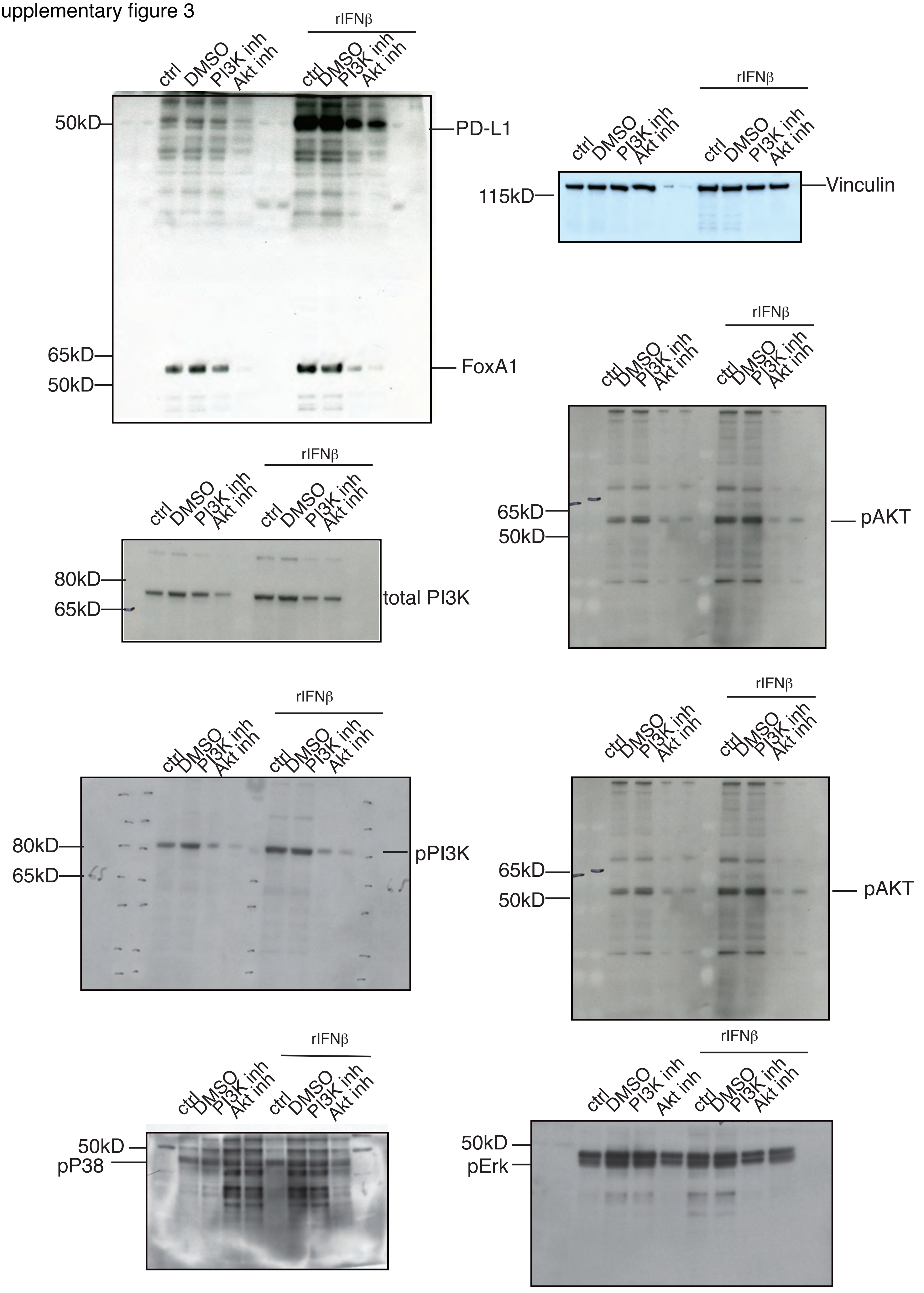

Supplementary figure 3: whole images of western blots in fgure 5f

Supplementary figure 4

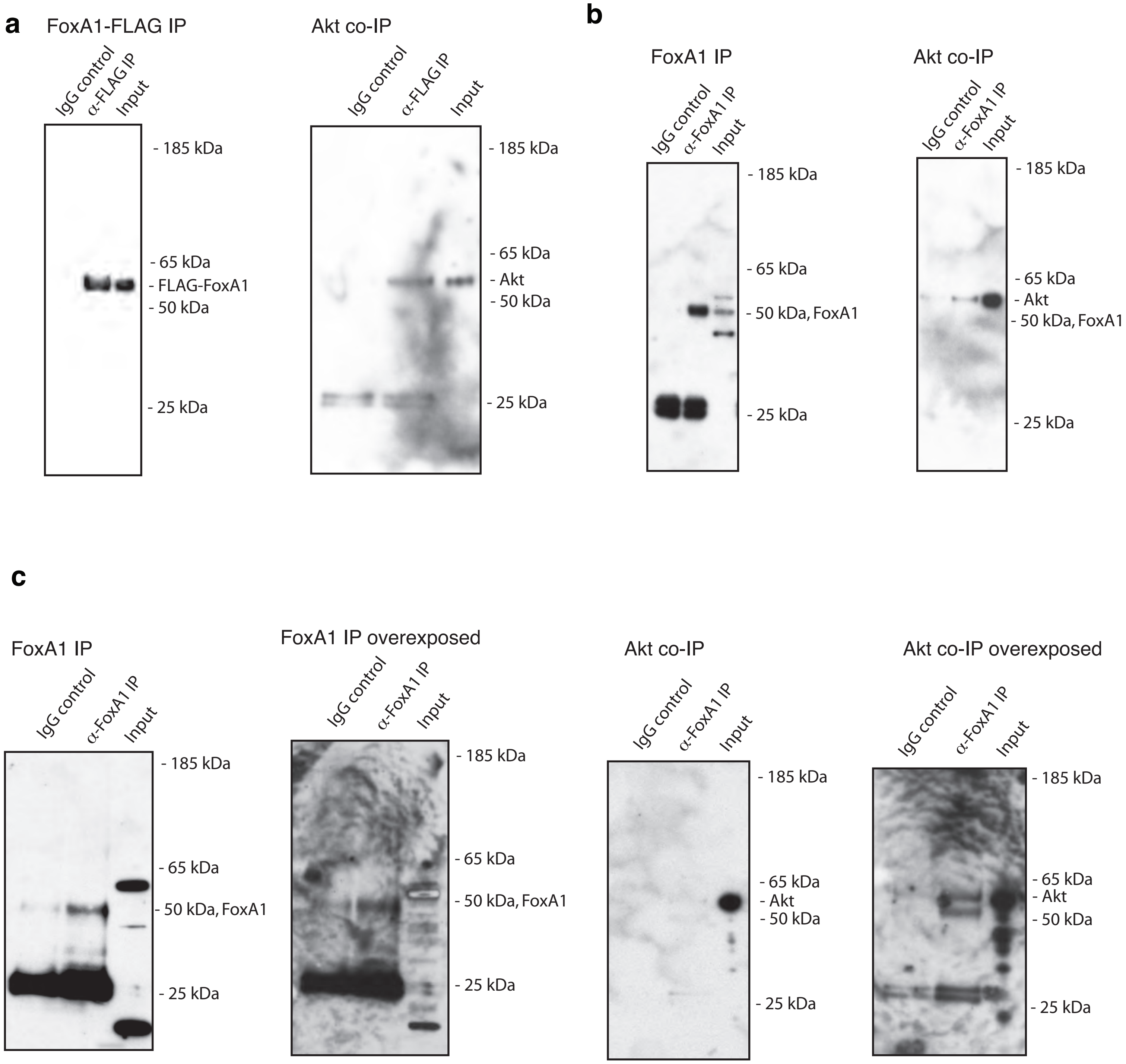

Supplementary figure 4: whole images of western blots in fgure 5

a) Figure 5i **b)** Figure 5j **c)** Figure 5k
